# Efficacy of sorafenib in patients with hepatocellular carcinoma after resection: a meta-analysis

**DOI:** 10.18632/oncotarget.21299

**Published:** 2017-09-28

**Authors:** Jin Shang, Shanling Xu, Jiaxing Zhang, Xuting Ran, Lang Bai, Hong Tang

**Affiliations:** ^1^ Center of Infectious Diseases, West China Hospital, Sichuan University, Chengdu, Sichuan, China; ^2^ Sichuan Cancer Hospital and Institute, Sichuan Cancer Center, School of Medicine, University of Electronic Science and Technology of China, Chengdu, China; ^3^ Chinese Evidence-based Medicine Center, Sichuan University, Chengdu, China; ^4^ West China Medical School, Sichuan University, Chengdu, Sichuan, China

**Keywords:** hepatocellular carcinoma, liver resection, sorafenib, overall survival, recurrence

## Abstract

**Background:**

The prognosis of hepatocellular carcinoma remains poor even after curative resection and it has no effective adjuvant therapy.

**Aim:**

This meta-analysis aimed to assess efficacy of sorafenib as adjuvant therapy for patients with hepatocellular carcinoma after resection.

**Materials and methods:**

A systematic search was conducted of Medline, Embase, Web of Science, Cochrane Library, Chinese Wanfang database, Chinese biological and medical database, China National Knowledgeand the Internet, data from 5 studies that included 296 participants were analyzed. The primary outcome was overall survival. Secondary outcomes included recurrence rate and mortality rate.

**Results:**

In the comparison of sorafenib versus control, no significant difference in overall survival (hazard ratio 1.39, 95% confidence interval [CI] 0.71–2.74, *P* = 0.34) or recurrence rate [risk ratio (RR) 0.81, 95% CI; 0.65–1.01, *P =* 0.06) was found. For mortality rate, subgroup analysis was conducted according to study type, only in subgroup 2, the RR was significantly reduced (0.66, 95% CI; 0.51–0.87, *P* = 0.003) in studies.

**Conclusions:**

In this meta-analysis, sorafenib achieves no significant benefit in any of the endpoints except a lower mortality rate in subgroup analysis, indicating that there is no convincing evidence of sorafenib as an effective adjuvant therapy in patients with hepatocellular carcinoma after resection.

## INTRODUCTION

Hepatocellular carcinoma (HCC) is the sixth most common solid cancer, causing 745,000 deaths worldwide annually, which ranks second as the cause of death among cancers. Moreover, recent data show an increasing trend in its incidence in many countries [1]. Most risk factors are known, such as chronic hepatitis B virus (HBV), chronic hepatitis C virus, alcohol consumption, nonalcoholic steatohepatitis, and diabetes [2]. Additionally, the development of a diagnosis technique of HCC by imaging or biopsy analysis enables to diagnose HCC larger than 10 mm [3]. However, the management of HCC remains a challenge.

Liver transplantation, liver resection, and liver ablation are considered potentially curative treatments. Liver resection applies to patients with early-stage HCC with preserved liver function, and it is the only option for large HCC but with preserved hepatic function. Moreover, recent research reports that liver resection, compared with ablation, is also the preferred management even for very early-stage small cancers [4]. Additionally, liver resection has some advantages over transplantation such as organ saving, lower costs, and lower dropout rate. However, the recurrence rate after liver resection is still as high as 70% at 5 years [5] with 5-year survival rates ranging from 60% to 80% [6, 7]. Thus, a high recurrence rate and unsatisfactory long-term prognosis of HCC highlight the need for adjuvant therapy.

Since HCC shows resistance to chemotherapy and radiotherapy, sorafenib, as the only standard oral multikinase inhibitor approved by the U.S. Food and Drug Administration and European Medicines Agency for HCC, has been demonstrated to benefit overall survival for HCC [8]. Also sorafenib has been proven to improve prognosis of advanced renal-cell carcinoma [9] and advanced thyroid cancer [10]. Furthermore, experiments *in vivo* show that sorafenib inhibits tumor growth and prevents metastatic recurrence after resection of HCC in nude mice [11]. However, recent opinions that sorafenib as adjuvant therapy could benefit patients with HCC after resection are still controversial [12–16].

To solve this problem, we conducted this meta-analysis to estimate the effectiveness of sorafenib as adjuvant therapy for HCC after liver resection.

## RESULTS

After screening and reviewing articles, five studies were selected. Characteristics of included studies are summarized in Table 1. Three of the studies were retrospective studies, one was an open-label controlled phase II trial, and one was a prospective controlled trial. All were conducted in patients with HCC after liver resection, and the dose of sorafenib varied from 200 mg to 800 mg/day. (Table 1).

**Table 1:** Characteristics of studies included in the meta-analysis

Authors (year)	Design	Population	Dose of sorafenib	Treatment duration	Follow-up time(range)	Outcomes	References
Wei Zhang (2014)	Retrospective with historical control	Patients with HCC after curative resection	400 mg twice daily	One month	–	Overall survival; recurrence rate and mortality rate	12
Shen-Nien Wang (2014)	Open-label, controlled phase II trail	Patients with HCC after curative resection	400 mg q.d	4 months	9.5-30.2 months	Recurrence rate; mortality rate	13
Lei Zhuang (2014)	Retrospective with historical control	Patients with HCC after curative resection	400 mg twice daily	–	13–44 months	Overall survival; mortality rate	14
Jiang Li (2016)	Retrospective with historical control	BCLC stage C HCC after curative resection	200–800 mg/d	One month	9–54 months	Overall survival; recurrence rate; mortality rate	15
Bingfeng Chen (2016)	Prospective controlled trail	Patients with HCC after curative resection	400 mg twice daily	6 months	36–60 months	Overall survival; recurrence rate; mortality rate	16

HCC: Hepatocellular carcinoma

Main characteristics of patients treated with sorafenib for HCC after liver resection are shown in Table 2. All included articles were published after 2014. A total of 109 patients were included, and the sample size varied from 12 to 32 patients, the range of median age was from 48.00 to 61.43, while the range of percentage of male patients was from 78.13% to 100%. The percentage of hepatitis B surface antigen positive rate ranged from 25% to 87.50%, and the mean tumor size range varied from 5.7 cm to 9.8 cm. (Table 2.)

**Table 2:** Characteristics of patients treated with sorafenib for patients with hepatocellular carcinoma after resection

Authors	Year	Region	Sample	Median Age	Males (%)	Hepatitis status (HBV/non-HBV)	Mean tumor Size (cm)	AFP(≧400 / < 400 ng/mL)	Cirrhosis(%)
Bingfeng Chen	2016	China	24	48.00	91.67%	–	–	-	62.5%
Jiang Li	2016	China	12	49.80	100.00%	3/9	9.8	8/4	0
Lei Zhuang	2014	China	27	48.19	92.59%	23/4	7.84	15/12	22.2%
Shen-Nien Wang	2014	China	14	61.43	92.86%	10/4	6.26	16/16(≧20/< 20 ng/mL)	0
Wei Zhang	2014	China	32	54.00	78.13%	28/4	5.7	–	–
Range	2014–2016	China	12–32	48.00–61.43	78.13%–100.00%	3–28/4–9	5.7–9.8	8–16/4–16	0–62.5

HBV: hepatitis B virus

### Effects of sorafenib on outcome

**Figure 1:**
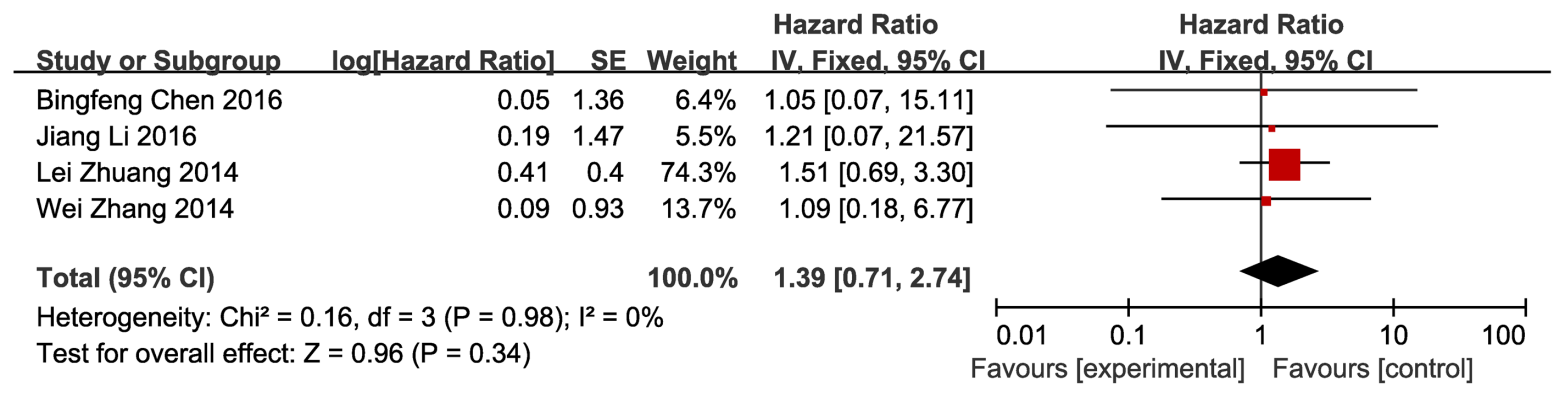
Efficacy of sorafenib for overall survival Shows forest plot of hazard ratio of overall survival of patients with hepatocellular carcinoma after resection who treated with sorafenib versus control Data of 4 studies were pooled using fixed-effects model. Studies are sorted by publication year.

Regarding overall survival as a primary outcome, four articles were reviewed and analyzed. After we pooled the data, adjuvant therapy using sorafenib for HCC after resection was prone to achieve longer overall survival with the hazard ratio of 1.39, but the 95% CI of hazard ratio was 0.71 to 2.74, *P* = 0.34, which showed that the benefit did not have statistical significance; with regard to heterogeneity analysis, both Cochran’s *Q* statistic and I^2^ showed no statistical significance. (Figure 1).

**Figure 2:**
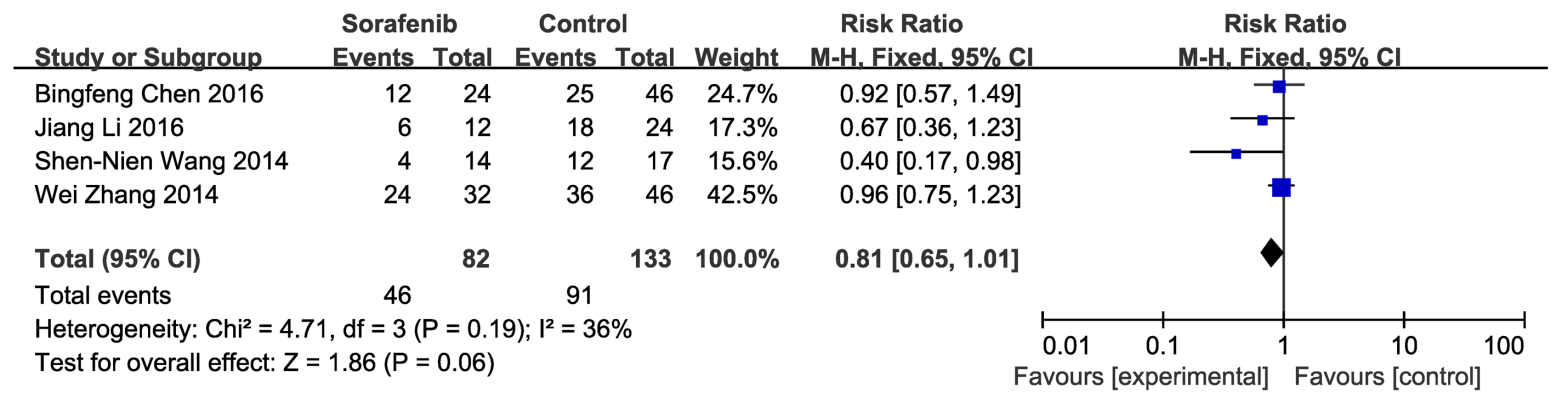
Efficacy of sorafenib for recurrence rate Shows forest plot of risk ratio of recurrence rate of patients with hepatocellular carcinoma after resection who treated with sorafenib versus control Data of 4 studies were pooled using fixed-effects model. Studies are sorted by publication year.

Secondary outcomes included recurrence rate and mortality rate. Four articles were analyzed, and data about recurrence rate were pooled: sorafenib versus control for HCC after resection was prone to achieve lower recurrence rate; however, the risk ratio was 0.81, and the 95% CI was 0.65 to 1.01, with no statistical significance(*P* = 0.06). With regard to heterogeneity analysis, both Cochran’s *Q* statistic and I^2^ showed no statistical significance. (Figure 2).

**Figure 3:**
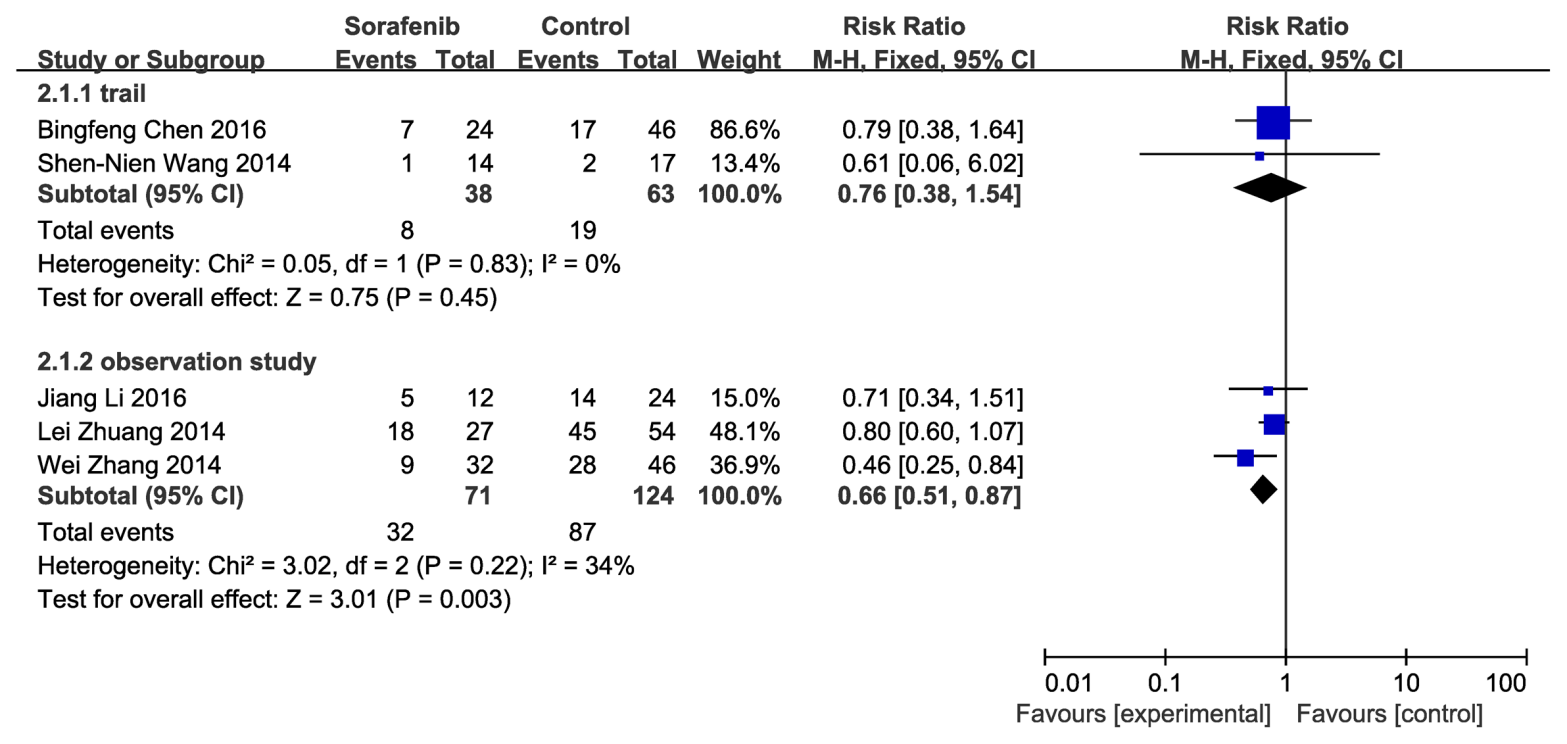
Efficacy of sorafenib for mortality rate Shows forest plot of risk ratio of mortality rate of patients with hepatocellular carcinoma after resection who treated with sorafenib versus control. Subgroup analysis of 5 studies according to study typewere conducted because of the high heterogeneity when pooling all data. Data of each subgroup were pooled using fixed-effects model. Studies are sorted by publication year.

With regard to mortality rate, Cochran’s *Q* statistical analysis showed df = 4 (*P* = 0.04) and I^2^ = 60%, which indicated high heterogeneity; after analysis of factors causing heterogeneity, subgroup analysis was conducted according to study design, and the heterogeneity of each group was then found to be low. Sorafenib for patients with HCC after resection achieved low mortality rate, with a risk ratio of 0.76 (CI 95% 0.38–1.54, no statistical significance) in clinical trials group, while the risk ratio was 0.66 (CI 95% 0.51–0.87, *P* = 0.003), with statistical significance in the observation studies group. (Figure 3).

Sensitivity analyses of all outcomes were conducted. No reverse emerged when trimming one study at a time, indicating the results were stable.

Adverse events related to sorafenib occurred at a high rate, with hand-foot skin reaction, diarrhea, alopecia, and hypertension as the most common side-effects. All patients recovered and no grade 4 adverse events or drug-related deaths occurred. No patients withdrew from the treatment of sorafenib (Table 3).

**Table 3:** Sorafenib-related adverse events

	Bingfeng Chen (2016)	Jiang Li (2016)	Lei Zhuang (2014)	Shen-Nien Wang (2014)	Wei Zhang (2014)
Sample^*^	24	12	27	14	32
Dose reduction	–	0	4	0	2
Treatment discontinuation	0	0	0	0	0
Hand-foot skin reaction	10	11	–	14	–
Diarrhea	7	10	–	8	–
Alopecia	9	0	–	14	–
Rash	6	0	–	1	–
Hypertension	5	10	–	3	–
Anorexia	14	0	–	3	–
Fatigue	12	0	–	3	–
Grade 3 adverse events^#^	–	–	6	0	6^Δ^
Hand-foot skin reaction	–	–	4	–	–
Diarrhea	–	–	2	–	–
Grade 4 adverse events^&^	–	–	0	0	0
Occurrence rate of grade 3 or worst Adverse events	–	–	0.22	0	0.19

*Sample means number of patients treated with sorafenib in publications.

- means data were not reported in included publications.

#Grade 3 Rash: hand-foot skin reaction is defined as ulcerative dermatitis or skin changes with pain interfering with function, Grade 3 diarrhea is defined as increase of ≥ 7 stools per day over baseline; incontinence; IV(intravenous) fluids ≥ 24 hours; hospitalization; severe increase in ostomy output compared to baseline; inferring with activities of daily living. ^&^Grade 4 diarrhea is defined as life-threatening consequences. ^Δ^ Not mentioned specifically.

## DISCUSSION

This is the first meta-analysis on sorafenib as adjuvant therapy for HCC after resection. The meta-analysis included a diverse population from different regions, though sorafenib was prone to achieve better outcomes, including longer overall survival, lower risk ratio, and lower mortality rate; however, only a significant lower mortality rate was found in observation studies through subgroup analysis, no statistically significant difference was found in other outcomes.

Adjuvant therapy is necessary for reducing recurrence rate of HCC after resection. However, there is no standard adjuvant therapy recommended yet, only interferon therapy showed its benefit but its side effect is nonnegligible. Current studies were mainly conducted on sorafenib monotherapy, there is no study on combination of sorafenib with other treatment as adjuvant therapy after liver resection yet and this could be a promising way in the future. Concerning sorafenib as monotherapy for HCC after liver resection, theoretically, it is worth investigating its adjuvant use to prevention of HCC recurrence for the reason that activation of the epidermal growth factor receptor, platelet-derived growth factor/receptor contributes to carcinogenesis, which is mediated by signaling pathways of angiogenesis and cell proliferation such as Ras/Raf/MEK/ERK [17]. Sorafenib as a multitargeted inhibitor was proven to inhibit them [18] and thought to be potentially effective in an adjuvant setting for HCC after resection. Contrary to theories, despite the effect of suppressing tumor and anti-angiogenic in advanced HCC, sorafenib did not show any benefit as an adjuvant therapy for HCC after resection.

These findings are similar to those of adjuvant sorafenib for other tumors such as renal carcinoma [19] and breast carcinoma [20]. Moreover, in patients with resected renal cell carcinoma, the addition of sorafenib shows substantial treatment discontinuation because of drug toxicity that grade 3 or worst adverse events were reported in 72% of patients treated with sorafenib. However, in our study, an occurrence rate of grade 3 or worse adverse events range from 0 to 0.22, only six patients received reduced sorafenib, and no patients withdrew from sorafenib treatment, which indicates that sorafenib was generally safe in patients with HCC after resection; thus, our study results were not affected.

With regard to sorafenib as adjuvant treatment, the unsatisfied efficiency is mainly attributed to the following: First, HBV DNA level was one of the most important acknowledged risk factors for postoperative HCC recurrence [21]. Antiviral treatment after resection is essential for either survival benefit or reducing recurrence [22]. In our study, four included articles reported the proportion of HBV-related HCC. However, the use of antiviral therapy was not elaborated. Therefore, it is uncertain whether efficiency of sorafenib was affected by HBV status. In other words, if patients with HBV-related HCC are not treated with antiviral therapy after resection, viral replication reaction and even liver failure is likely to occur.

Regarding other risk factors associated with survival of HCC after resection, microvascular invasion, tumor volume, platelet count, serum albumin, and sex have been reported to date [23]. Among these risk factors, microvascular invasion was considered the most important predictive factor for survival [24]. In addition, microvascular invasion is also an independent risk factor for early recurrence after resection [25]. Early recurrence and late recurrence are two different patterns. The important risk factor for late recurrence is cirrhosis [26]. Consequently, the issue was whether sorafenib as an adjuvant treatment could affect microvascular invasion and cirrhosis to benefit patients with HCC after resection.

Microvascular invasion (MVI) is mediated by a complex course called epithelial-mesenchymal transitions (EMT). EMT has been validated in HCC progression [27]. In brief, tumor cells acquire mesenchymal markers and produce specific proteases, and the specific proteases then lead to the degradation of extracellular matrix; further, the cell-to-cell adhesion decreases and apico-basal polarity losses facilitate the invasion [28]. Consequently, once EMT occurs, many pathways and molecules, such as E-cadherin, cytokeratins, Wnt pathway, and Notch pathway, lead to sorafenib resistance [29, 30]. Thus, the relationship between EMT and sorafenib resistance can explain why sorafenib did not benefit patients with MVI. Moreover, even though sorafenib can suppress tumor proliferation and angiogenesis mainly mediated by inhibiting Raf and VEGFR(vascular endothelial growth factor receptor), no evidence exists that it can prevent MVI. On the contrary, Raf inhibitor activated the ERK cascade signal [31], and VEGFR inhibitor accelerate the metastasis [32]. Whether the effects of sorafenib can sustain the progression need further confirmation. With regard to the other risk factor, cirrhosis, sorafenib could neither improve liver function nor treat cirrhosis. Consequently, the invalidity of sorafenib on the two main risk factors could explain the results of our study.

When comparing adjuvant sorafenib therapy to other curative treatments of HCC, it was reported that sorafenib is associated with an acceptable safety profile and survival benefit in patients with HCC suffering recurrence after liver transplantation [33]. Sorafenib might also benefit patients with HCC after resection; nevertheless, the status of hepatocyte, risk factors of recurrence, or prognostic factors are different. Hallmarks that include sustaining proliferative signaling, evading growth suppressors, and tumor-promoting inflammation, have changed in patients with HCC despite the resection [34].

In conclusion there were no significant differences in any of the endpoints except a lower mortality rate in subgroup analysis, indicating that this meta-analysis provides no convincing evidence that sorafenib is an effective adjuvant therapy in patients with HCC after resection. However, more prospective studies are required in the future, allowing this meta-analysis to be updated.

## MATERIALS AND METHODS

### Selection of trials

**Figure 4:**
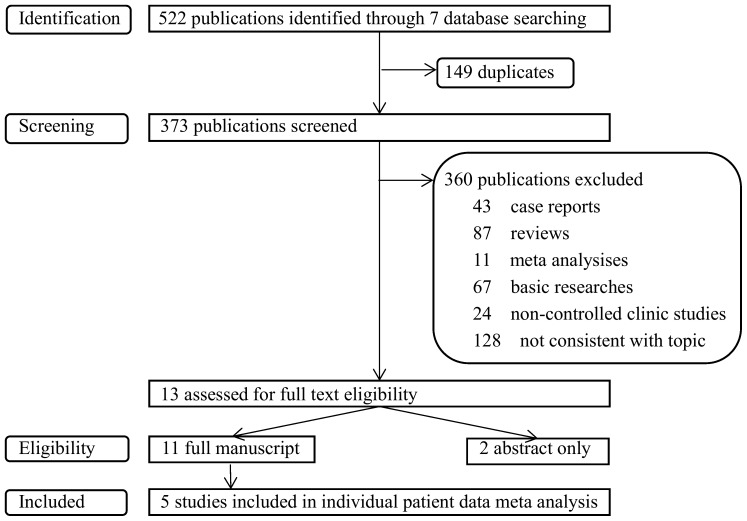
Flow diagram of studies indentified, screened, included and excluded Shows literature research and selection strategy following prisma flow diagram 4 steps were conducted including identification, screening, eligibility, included. Inapplicable publications were excluded in each step and 5 publications were included at last.

This analysis was conducted according to the PRISMA (Preferred Reporting Items for Systematic Reviews and Meta-Analyses) statement. Publications were screened and identified through a search of Medline, Embase, Web of Science, Cochrane Library, Chinese Wanfang database, Chinese biological and medical database, China National Knowledge and the Internet. The search strategy was that patients (with HCC after liver resection) and intervention (sorafenib) searches were not limited by date but by English and Chinese language. Then, the title and abstract were screened, and the retrieved articles were further analyzed for their reference lists. Included criteria were full-length articles regarding the use of sorafenib for treatment of HCC after liver resection, including patients with HCC after liver resection. Case reports, ongoing studies, and articles with incomplete information were excluded. The primary outcome was overall survival. Secondary outcomes included recurrence rate and mortality rate. Among the 522 studies reviewed (Figure 4), 13 articles were selected, including 11 full-length articles. However, this meta-analysis includes only five full papers because the other six papers [35–40] did not regard selective outcome or possessed incomplete information.

### Review of trials

Three independent investigators reviewed and evaluated the retrieved articles about patient characteristics, interventions, outcomes, and study validity. Controversies among investigators were not common and were solved by discussion or counseling evidence-based medicine experts. Quality of evidence for randomized studies was assessed according to the Cochrane Collaboration’s tool for assessing risk of bias [41], and the Newcastle Ottawa Scale was used to assess quality of nonrandomized studies [42].

### Statistical analysis

Summary hazard ratios, risk ratios, and 95% CIs were calculated using fixed effects models or random effects models based on heterogeneity and Cochran’s Q statistic, and I^2^was used to assess the heterogeneity across studies. Subgroup analysis stratified by study design was conducted. A *p*-value < 0.05 was considered statistically significant. The sensitivity of meta-analysis was assessed by trimming one study, and the pooled results were then compared before and after the trimming. All analyses were performed by Review Manager Software.

## Abbreviations

HCC: hepatocellular carcinoma
HBV: hepatitis B virus
PRISMA: Preferred Reporting Items for Systematic Reviews and Meta-Analyses
CI: confidence interval
